# Effects of Deep Shading on Agronomic Traits, Coloration, and Antioxidant Properties in Sweetpotato Leaves

**DOI:** 10.3390/plants14192969

**Published:** 2025-09-25

**Authors:** Yang Lu, Jian Wang, Yizhao Chen, Jingjing Li, Zengrui Li, Sunjeet Kumar, Zhixin Zhu, Yong-Hua Liu, Guopeng Zhu

**Affiliations:** 1 School of Breeding and Multiplication (Sanya Institute of Breeding and Multiplication), Hainan University, Sanya 572025, China184224@hainanu.edu.cn (S.K.); 2 Key Laboratory for Quality Regulation of Tropical Horticultural Crops of Hainan Province, School of Tropical Agriculture and Forestry, Hainan University, Haikou 570228, China

**Keywords:** vegetable sweetpotato, leaf color, chlorophylls, anthocyanins, antioxidant capacity, soluble sugar

## Abstract

The vegetable sweetpotato (*Ipomoea batatas* L.) is a novel, specialized type, cultivated for its tender stems and leaves, which are rich in nutrients and bioactive compounds. To clarify its growth adaptation to weak light conditions often encountered during cultivation, this study examined the impact of 70% shading on 12 representative cultivars from 4 leaf color types. Agronomic traits, color, and nutritional and antioxidant properties were assessed in both young and mature leaves. Shading promoted leaf expansion, plant height, and vine length, but reduced stem thickness and dry-matter content. Leaf shape shifted from lobed to more cordate, with the foliage becoming darker green and lighter red due to elevated chlorophylls and reduced anthocyanins. Shading generally reduced soluble protein, sugar, cellulose, total phenols, flavonoids, and total antioxidant capacity. Antioxidant capacity correlated most strongly with soluble sugar and dry-matter content, followed by total phenols and flavonoids. Gene expression analysis of key light- and leaf color-related genes revealed up-regulation of chlorophyll genes and down-regulation of anthocyanin genes under shading, with light-responsive genes potentially affected by pigment feedback. These results elucidate the sweetpotato’s adaptive responses to deep shading and provide valuable guidance for optimized cultivation and breeding of vegetable sweetpotato in light-limited environments.

## 1. Introduction

Sweetpotato (*Ipomoea batatas* L.) is a crucial crop in Asia, where traditional varieties are primarily cultivated for their storage roots [1]. While the stems and leaves are also edible, their coarse texture has limited culinary applications, often restricting their use to occasional livestock feed [2]. In recent decades, novel varieties bred specifically for tender stems and leaves—known as vegetable sweetpotato—have gained popularity. Notable examples include cultivars such as ‘Fu 18’ and ‘Shulv No.1’ [3,4].

Vegetable sweetpotato serves as an alternative to leafy greens during summer shortages. Its leaves and stems are rich in minerals, vitamins, and various bioactive compounds, which contribute to high antioxidant activity and significant health benefits [5,6,7]. Key bioactive constituents include phenolic acids (e.g., chlorogenic acids and caffeic acid derivatives), flavonoids (e.g., luteolin, quercetin, apigenin, and anthocyanins), galactolipids, carotenoids, dietary fiber, dietary protein, and polysaccharides [6]. These metabolites not only enhance the plant’s resistance to stress but also offer multiple health-promoting effects for humans, including antioxidative, antimutagenic, hepatoprotective, and hypoglycemic properties [6,8,9]. Regular consumption of vegetable sweetpotato has been associated with reduced cancer risk [10]. And they are regarded as “anti-cancer vegetables” in the medical community, “longevity vegetables” in Japan, and “queen of greens” in Taiwan and Hong Kong [3].

Light serves both as the energy source for photosynthesis and an environmental signal regulating plant growth and physiology [11]. Energetically, plants utilize photosynthetic pigments (chlorophylls and carotenoids) to capture light for carbon assimilation. As a signal, light is perceived through photoreceptors—including receptors of red light, blue light, and UV-B—triggering adaptive responses in growth and development [11]. Light intensity profoundly influences plant growth, with both excess and insufficient light adversely affecting plant performance [11]. For instance, tea leaves under high light showed chlorophyll degradation, reduced antioxidant enzyme activity, and elevated soluble sugar and protein levels [12]. In spinach, the contents of soluble sugar, protein, and vitamin C increased with light intensity from 120 to 300 µmol·m^−2^·s^−1^ [13].

Vegetable sweetpotato, a light-loving crop typically cultivated in summer, is often exposed to intense sunlight. The effects of shading on its growth and quality remain poorly understood. Meanwhile, modern agricultural systems—such as intercropping, plant factories, and soilless cultivation—often reduce the availability of natural light, subjecting crops to prolonged low-light stress. Although previous studies have examined the influence of light intensity on sweetpotatoes, most have focused on underground storage roots [14,15]. For instance, metabolomic profiling showed increased flavonoids and numerous other metabolites in the roots of purple-fleshed sweetpotato ‘Jihei-1’ under 60% shading [14]. Similarly, research on ‘Ziluolan’ indicated that 20% shading enhanced sugar content in tubers, though greater shading reduced the dry matter of the whole plant [15]. Studies on the leafy variety ‘Eshu10’ revealed a positive correlation between light intensity and yield, and shading reduced photosynthesis and decreased soluble sugar, starch, and sucrose content [16].

Current research on the effects of shading on the aerial parts of sweetpotatoes is limited to one or two varieties and mainly focuses on photosynthetic performance. Responses in aboveground tissues—particularly across diverse varieties—remain poorly understood. To bridge this gap, we systematically evaluated the impact of shading on the aerial parts of 12 representative sweetpotato cultivars, encompassing four distinct leaf color types: green, yellow, red-to-green, and red. Under 70% shading, we analyzed changes in agronomic traits, pigment contents, physiological properties, and the expression of key genes related to leaf color and light adaptation in both young and mature leaves. This study aims to clarify how vegetable sweetpotato adapts to low-light conditions and to identify shade-tolerant cultivars. Our findings advance the understanding of phenotypic plasticity in vegetable sweetpotato and provide practical guidance for optimized cultivation and breeding in light-limited environments.

## 2. Results

### 2.1. Agronomic Traits of Sweetpotato Aboveground Parts in Response to Deep Shading

Twelve sweetpotato varieties were evaluated under 70% shading (Table 1).

Vegetable sweetpotato varieties have been developed from the common sweetpotato by selecting those with tender, palatable stems and leaves. Here, we selected 12 representative varieties with acceptable palatability and distinct leaf characteristics, covering a wide range of leaf shapes, colors, and usage types (Table 1, Figure S1).

Among the 12 varieties, leaf shapes ranged from cordate (e.g., ‘Chuzi1’ and ‘Fu23’) to deeply lobed (e.g., ‘HD-V7’). Based on usage, the varieties included five vegetable types, four tuberous root types, one ornamental type, and two dual-usage types (‘HD-V7’ and ‘HD7791’). In terms of leaf color, they were classified into four categories: Green (‘Fu18’, ‘HD-V4’, ‘HD-V7’, and ‘RXC1’), Yellow (‘Golden’), Red to Green (‘Ayamurasaki’, ‘Su24’, ‘Fu202’, and ‘HD7791’), and Red (‘Chuzi1’, ‘GCS12-557’, and ‘Fu23’) (Figure S1). “Red to Green” varieties have red young leaves and green mature leaves, while “Red” varieties typically feature greenish apical tops (Figure S2). These color variations reflect divergent light adaptation and pigment metabolism strategies and served as the primary classification criterion in this study.

Shading significantly altered the aboveground agronomic traits across all varieties (Table 1). Compared to natural light conditions (CK), plants under 70% shading exhibited increased leaf area, plant height, vine length, and internode length, while stem thickness and dry matter content generally decreased. Specifically, leaf area increased from 29.14–68.57 cm^2^ to 37.32–80.72 cm^2^; plant height from 13.72–24.61 cm to 17.18–28.73 cm; vine length from 21.17–54.12 cm to 32.19–62.57 cm; and internode length from 1.33–4.59 cm to 1.96–5.60 cm. In contrast, dry matter content decreased from 10.08–14.35% to 8.45–12.01%, and stem diameter declined from 4.03–6.97 mm to 3.68–5.73 mm. Branch number decreased in eight varieties and increased in two, with significant changes only in ‘Fu18’ (50% reduction, **) and ‘Su24’ (184% increase, *).

The extent of change varied notably among cultivars. ‘Su24’ showed the largest increases in leaf area (168.3%), vine length (188%), branch number (184%), and internode length (169%), though its dry matter content was unaffected. ‘Fu18’ exhibited moderate changes in most traits, but the greatest reduction was in branch number. Overall, no clearly shade-tolerant cultivar could be identified based on these trait alterations.

### 2.2. Morphological and Color Changes in Sweetpotato Leaves in Response to Deep Shading

#### 2.2.1. Effects of Shading on Leaf Morphology and Color

**Figure 1:**
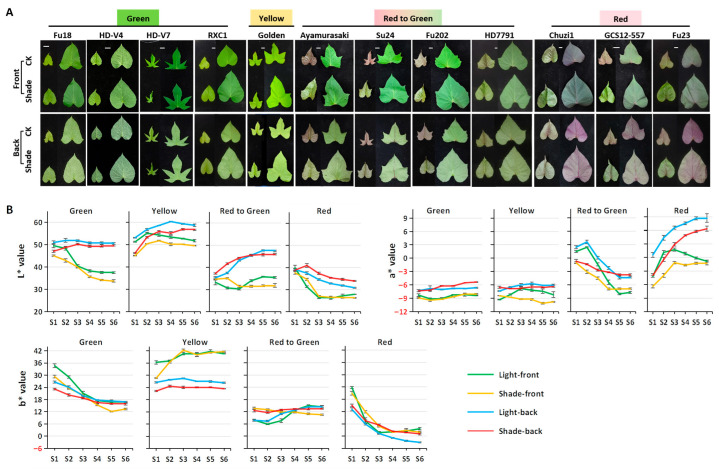
Effects of deep shading on leaf morphology and color in sweetpotato varieties. (**A**) Representative leaves at stages S2 and S5 under natural light (CK) and 70% shading. Bar = 1 cm. (**B**) CIEL*a*b* parameters for leaves from S1 to S6 for sweetpotato varieties from four color series. The front represents the adaxial surface of the leaves, and the back represents the abaxial surface.

Shading promoted leaf enlargement in all varieties (Table 1, Figure 1A and Figure S3). While two varieties (‘Chuzi1’ and ‘Fu23’) maintained a stable cordate shape under both light conditions, others developed deeper lobes under full light that were significantly reduced under shading. For instance, ‘Fu18’ exhibited reniform leaves with one or more lobes under full light but produced a higher proportion of cordate leaves under low light. These results suggest that high light promotes lobing, whereas shading leads to rounder, less-lobed leaves in sweetpotato.

Shading visibly intensified the green hues in the “Green” and “Yellow” groups, while reducing the red hues in the “Red to Green” and “Red” groups. For red leaves, the red color on the adaxial surface became less vivid, and the purple hues on the abaxial surface lightened. The typical red hue of young leaves in “Red to Green” varieties became less distinct under shaded conditions (Figure 1A and Figure S3).

#### 2.2.2. Effects of Shading on Leaf Color Parameters

Color parameters (L*, a*, and b*) were measured across leaf developmental stages S1–S6 (Figure 1B and Figure S4). L* indicates lightness (0–100), a* represents green (−) to red (+), and b* indicates blue (−) to yellow (+). 

L* was highest in the yellow variety ‘Golden’. Abaxial surfaces had higher L* across all varieties, consistent with their whitish appearance. Shading decreased L* in green/yellow leaves but increased L* in red leaves. For example, in “Red to Green” varieties, L* was higher under shading during S1–S3 (red phase) but was lower at S4–S6 (green phase).

The a* values (positive for red, negative for green) aligned well with observed leaf color. Adaxial surfaces generally had a lower a* than abaxial surfaces. Shading reduced a* in red-hued groups. Green/Yellow varieties maintained stable negative a* values, while red-hued groups showed considerable variation during development. In “Red to Green” varieties, a* decreased until stabilizing at S5–S6, reflecting red-to-green leaf color transition. In “Red” varieties, abaxial a* increased, while adaxial a* rose at S1–S3 and declined at S4–S6, likely due to pigment dilution during leaf expansion.

The b* values were notably higher on the adaxial side in ‘Golden’, but were similar among the four surfaces of other color groups. During leaf development, b* declined with maturation in “Green” and “Red” varieties, and turned negative in “Red” leaves at S4–S6 under CK. Shading decreased b* in green leaves but increased b* in red leaves.

In summary, 70% shading reduced L* and b* in green leaves, increased them in red leaves, and decreased a* across varieties, consistent with the observed darkening of green leaves and lightening of red leaves.

#### 2.2.3. Effects of Shading on Leaf Pigment Contents

Leaves from S1 to S3 were pooled as young leaves (Y), and S4–S6 as mature leaves (M), under both natural light (CK) and 70% shading conditions across 12 sweetpotato varieties (Figure S2). Each variety, therefore, included four sample types: young leaves under natural light (YL), young leaves under shading (YS), mature leaves under natural light (ML), and mature leaves under shading (MS).

**Figure 2:**
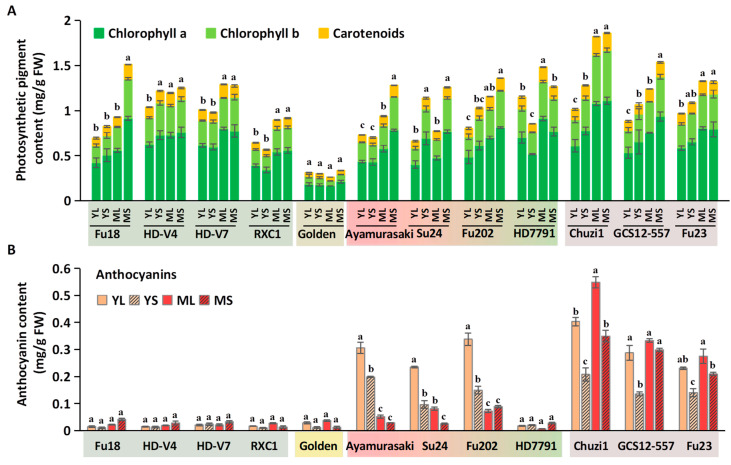
Effects of deep shading on contents of photosynthetic pigments and anthocyanins in sweetpotato leaves. (**A**) Photosynthetic pigment contents, including chlorophyll a, b, and carotenoids. (**B**) Anthocyanin contents. Significant differences are denoted by letters for the four sample types (YL, YS, ML, and MS) of each cultivar (one-way ANOVA, n = 3, *p* < 0.05).

Sweetpotato leaves contain two major classes of pigments: photosynthetic pigments (chlorophyll a, b, and carotenoids) and anthocyanins [17]. The contents of chlorophyll a, chlorophyll b, and carotenoids were highly correlated, with an average ratio of 5.4:2.5:1, and showed nearly identical trends across the four tissue types within each variety. Thus, their combined levels are presented in Figure 2A. Among the 12 varieties, ‘HD7791’ had the highest photosynthetic pigment content among young leaves, while ‘Chuzi1’ ranked highest in mature leaves. ‘Golden’ exhibited the lowest pigment levels, with no significant differences among its four sample types. In the remaining 11 varieties, mature leaves contained higher pigment levels than young leaves, and 70% shading further promoted pigment accumulation.

Anthocyanins were mainly detected in red-hued varieties (Figure 2B). Within the “Red to Green” group, ‘HD7791’ had relatively low anthocyanins, while the other three varieties showed comparable levels. Anthocyanins were highest in YL and decreased with both shading and maturation. In the “Red” group, mature leaves contained more anthocyanins than young leaves, and shading reduced anthocyanin content. ‘Chuzi1’ had the highest anthocyanin content, followed by ‘GCS12-557’ and ‘Fu23’.

In summary, shading increased chlorophyll and carotenoid content but decreased anthocyanins. These shifts align with the observed leaf color phenotypes (Figure 1A) and color parameters (Figure 1B): high photosynthetic pigment levels correlate with lower a* values, while anthocyanin elevates a* values.

### 2.3. Changes in Nutritional and Antioxidant-Related Physiological Indices

**Figure 3:**
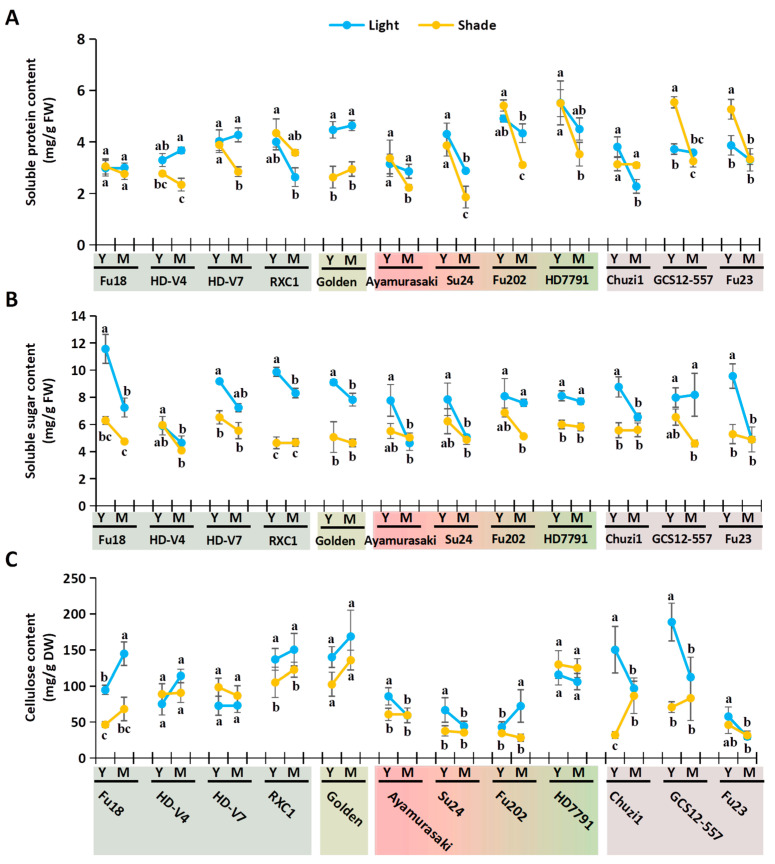
Effects of deep shading on nutritional indices in young and mature sweetpotato leaves. (**A**) Soluble protein contents; (**B**) Soluble sugar contents; (**C**) Cellulose contents. Y, young leaves; M, mature leaves. Significant differences are denoted by letters for the four sample types of each cultivar (one-way ANOVA, n = 3, *p* < 0.05).

**Figure 4:**
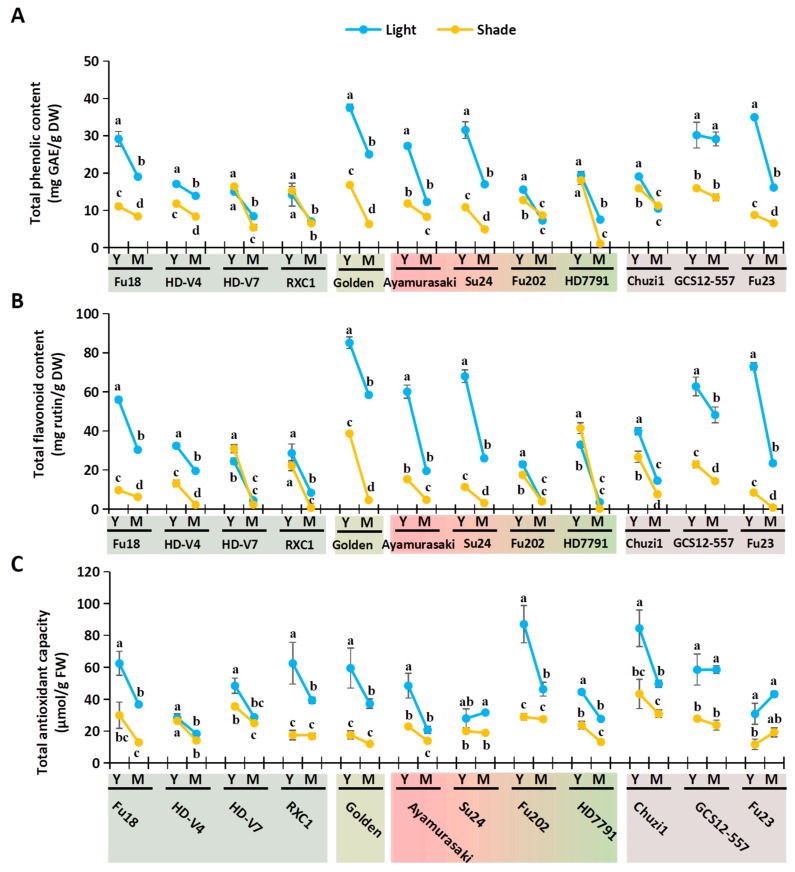
Effects of deep shading on antioxidant-related indices in young and mature sweetpotato leaves. (**A**) Total phenolic contents; (**B**) Total flavonoid contents; (**C**) Total antioxidant capacities. Y, young leaves; M, mature leaves. Significant differences are denoted by letters for the four sample types of each cultivar (one-way ANOVA, n = 3, *p* < 0.05).

Sweetpotato leaves are valued as a nutritious and antioxidant-rich functional vegetable [2,6]. We analyzed key nutritional and antioxidant-related physiological indices in the four sample types (YL, ML, YS, and MS) across 12 varieties. The nutritional indices assessed included soluble protein, soluble sugar, and cellulose (Figure 3), while antioxidant-related indices comprised total phenolic content (TPC), total flavonoid content (TFC), and total antioxidant capacity (Figure 4). In the dot-line plots, blue represents natural light (L) and yellow denotes shading (S); solid lines connect data points from young to mature leaves within each variety. The overall effects of shading and leaf development can be visually inferred from the distribution and trends of the blue and yellow lines.

Soluble protein content ranged from 1.86 to 5.54 mg/g FW (Figure 3A). Light-exposed leaves averaged 3.75 mg/g FW vs. 3.49 mg/g FW under shading. Young leaves showed similar average levels between light (YL: 4.01 mg/g FW) and shade (YS: 4.07 mg/g FW), but mature shaded leaves (MS: 2.90 mg/g FW) had lower protein than light-grown mature leaves (ML: 3.50 mg/g FW), indicating that shading reduced protein mainly in mature leaves. Eight varieties had higher protein under light, while four varieties (RXC1, Chuzi1, GCS12-557, and Fu23), mainly “Red” varieties, showed the opposite. Most varieties declined in protein with maturation, except green/yellow varieties, which showed a non-significant increase. Only ‘Fu18’ (2.76–3.05 mg/g FW) showed no significant differences across treatments.

Soluble sugar content ranged from 4.09 to 11.57 mg/g FW (Figure 3B). Light-exposed leaves averaged 7.65 mg/g FW vs. 5.42 mg/g FW under shading. Both YL (8.65 mg/g FW) > YS (5.88 mg/g FW) and ML (6.66 mg/g FW) > MS (4.97 mg/g FW) indicated shading reduced sugar in young and mature leaves. All varieties had lower sugar under shade, except ‘HD-V4’. Sugar declined with maturation across all varieties. ‘Fu18’ YL had the highest content (11.57 mg/g FW), while ‘HD-V4’ YL was the lowest (5.90 mg/g FW). Among mature leaves, ‘RXC1’ ML was highest (8.31 mg/g FW), and ‘Ayamurasaki’ ML was the lowest (4.63 mg/g FW).

Cellulose content ranged from 28.38 to 188.73 mg/g DW (Figure 3C). Light-exposed leaves averaged 100.01 mg/g DW, compared to 75.32 mg/g DW under shading. Mean values across phenotypes—YL (102.30 mg/g DW), YS (71.13 mg/g DW), ML (97.73 mg/g DW), and MS (79.51 mg/g DW)—suggest shading reduced cellulose, though developmental effects were inconsistent. Ten varieties had higher cellulose under light; exceptions (‘HD-V7’, ‘HD7791’) were not significant. Green/yellow varieties trended upward with maturation (mature > young), while red-leaf varieties declined (young > mature). ‘GCS12-557’ YL had the highest value (188.73 mg/g DW), and ‘Fu202’ YS had the lowest (34.67 mg/g DW). Among mature leaves, ‘Golden’ ML was highest (168.70 mg/g DW) and ‘Fu23’ MS was the lowest (29.93 mg/g DW).

Many studies on the functional compounds of sweetpotato leaves have indicated that their health benefits are primarily associated with polyphenols and flavonoids [5,6]. Antioxidant indicators—TPC, TFC, and antioxidant capacity—showed highly consistent decreasing trends under shading and with maturation across nearly all varieties (Figure 4). With few exceptions, light-exposed leaves consistently outperformed shaded leaves (blue lines over yellow), and younger leaves exceeded mature leaves (lines trended downward).

TPC ranged from 1.11 to 37.54 mg GAE/g DW (Figure 4A). Light-exposed leaves averaged nearly double (19.35 mg/g DW) that of shaded leaves (10.62 mg/g DW). YL (24.26 mg/g DW) > YS (13.79 mg/g DW) and ML (14.44 mg/g DW) > MS (7.44 mg/g DW). ‘Golden’ YL had the highest TPC (37.54 mg/g DW), while ‘RXC1’ YL was the lowest (14.19 mg/g DW). Notably, shaded mature ‘HD7791’ leaves dropped to an extreme low (1.11 mg/g DW).

TFC varied from 0.21 to 85.14 mg rutin/g DW (Figure 4B). Light-exposed leaves averaged 35.29 mg/g DW—nearly triple that of shaded leaves (12.86 mg/g DW). YL (48.86 mg/g DW) far exceeded YS (21.71 mg/g DW), and ML (21.51 mg/g DW) significantly surpassed MS (4.21 mg/g DW). In mature leaves, flavonoid levels collapsed in some varieties (e.g., ‘RXC1’, ‘HD7791’, and ‘Fu23’), falling below 1 mg/g DW.

Antioxidant capacity aligned with TPC and TFC trends, except in ‘Fu23’, where mature leaves retained high activity despite low phenolics/flavonoids, likely due to the accumulation of anthocyanins (Figure 4C). ‘HD7791’ mature leaves maintained normal antioxidant levels despite having minimal phenolics/flavonoids, possibly owing to other antioxidants such as chlorogenic acids [6,7].

These results demonstrate that variety, maturity, and light conditions collectively shape the nutritional and antioxidant properties of sweetpotato leaves.

### 2.4. Correlation Between Agronomic, Nutritional, and Antioxidant Indices

**Figure 5:**
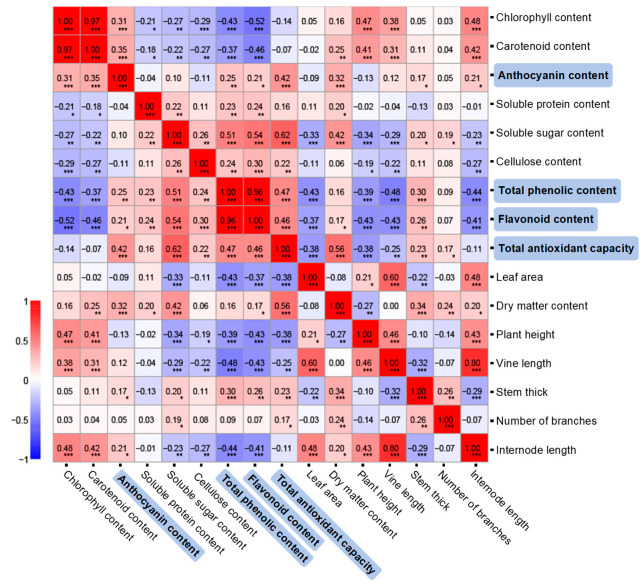
Pearson’s correlation heatmap of agronomic, nutritional, and antioxidant indices. Differences were significant at *p* < 0.05 (*), *p* < 0.01 (**), and *p* < 0.001 (***).

The correlation coefficients (R) were calculated among the 16 agronomic, nutritional, and antioxidant indices (Figure 5). Three index pairs showed highly significant positive correlations: chlorophylls and carotenoids (R = 0.97), TPC and TFC (R = 0.96), and vine length and internode length (R = 0.8). Among the three pairs, the TPC–TFC pair exhibited significant negative correlations with the other two pairs (ranging from −0.52 to −0.37). Other notable high positive correlations included a positive association between leaf area and vine length (R = 0.6).

Total antioxidant capacity correlated most strongly with soluble sugar content (R = 0.62), followed by dry-matter content (R = 0.56), TPC (R = 0.47), TFC (R = 0.46), and anthocyanins (R = 0.42). Negative correlations were observed for total antioxidant capacity with leaf area (−0.38), plant height (−0.38), and vine length (−0.25).

### 2.5. Expression of Key Leaf Color and Light-Related Genes in Sweetpotato Leaves

**Figure 6:**
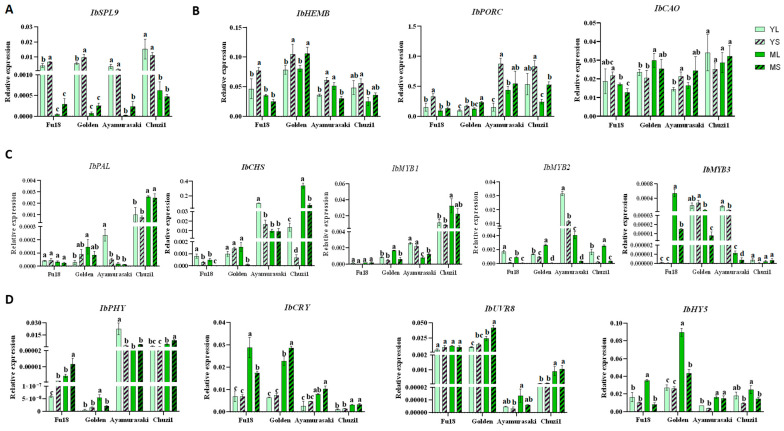
Expression of key genes related to leaf color and light responses. (**A**) Development-associated gene *IbSPL9*. (**B**) Genes involved in the chlorophyll biosynthetic pathway, including *IbHEMB*, *IbPORC*, and *IbCAO*. (**C**) Genes within the anthocyanin biosynthetic pathway and pivotal R2R3-MYB TFs, encompassing *IbPAL*, *IbCHS*, *IbMYB1*, *IbMYB2*, and *IbMYB3*. (**D**) Genes related to light signal transduction, including *IbPHY* (red light receptor), *IbCRY* (blue light receptor), *IbUVR8* (UV-B receptor), and *IbHY5* (a core TF in light signal transduction). Significant differences among the four sample types for each cultivar are indicated by letters (one-way ANOVA, n = 3, *p* < 0.05).

To explore the molecular mechanisms of shade-induced responses in sweetpotato leaves, four representative varieties with distinct leaf colors were selected: ‘Fu18’, ‘Golden’, ‘Ayamurasaki’, and ‘Chuzi1’ (Figure 6). We examined the expression of key genes involved in leaf development, chlorophyll biosynthesis, and anthocyanin biosynthesis, as well as components of the light signaling pathway. The genes analyzed were as follows: the development-associated gene *IbSPL9* (Squamosa Promoter Binding Protein-like 9) [18]; chlorophyll biosynthetic genes, including *IbHEMB* (Heme Biosynthesis Gene B), *IbPORC* (Protochlorophyllide Oxidoreductase C), and *IbCAO* (Chlorophyll a Oxygenase) [19]; genes within the anthocyanin biosynthetic pathway and pivotal R2R3-MYB transcription factors (TFs), comprising *IbPAL* (Phenylalanine Ammonia-Lyase), *IbCHS* (Chalcone Synthase), *IbMYB1*, *IbMYB2*, and *IbMYB3* [17,20]; and genes related to light signal transduction, including *IbPHY* (Phytochrome, red light receptor), *IbCRY* (Cryptochrome, blue light receptor), *IbUVR8* (UV Resistance Locus 8, UV-B receptor), and *IbHY5* (Elongated Hypocotyl 5, a core TF in light signal transduction) [21,22].

Expression of *IbSPL9* was higher in young leaves than in mature ones, consistent with its developmental role, and was largely unaffected by shading (Figure 6A). Among chlorophyll biosynthesis genes, *IbHEMB* expression was highest in ‘Golden’. Shading increased its expression in young leaves but induced variable responses in mature leaves. *IbPORC* expression was higher in red-leaf varieties than in yellow-green ones, and increased under shading across all varieties. *IbCAO* expression decreased in ‘Golden’ yet increased in ‘Ayamurasaki’ under shading; it increased in young leaves but decreased in mature leaves of ‘Fu18’, while the opposite pattern was observed in ‘Chuzi1’ (Figure 6B).

For anthocyanin-related genes, the expression of structural genes (*IbPAL* and *IbCHS*) and regulatory TFs (*IbMYB1*, *IbMYB2*, and *IbMYB3*) correlated with red pigmentation, and their downregulation under shading aligned with reduced redness (Figure 6C). Among the MYB activators, *IbMYB2* was the highest expressed in ‘Ayamurasaki’, followed by *IbMYB1*; in ‘Chuzi1’, *IbMYB1* predominated, followed by *IbMYB2*. The expression of *IbMYB3* was low across all varieties, suggesting variety-specific dependence on different MYB regulators for anthocyanin accumulation [23].

Among light-signaling genes, *IbPHY* and *IbUVR8* displayed contrasting cultivar-specific expression: *IbPHY* was upregulated in red-hued cultivars, while *IbUVR8* was higher in yellow/green varieties (Figure 6D). Although less cultivar-specific, *IbCRY* and *IbHY5* were significantly elevated in mature yellow/green leaves. Shading suppressed *IbHY5* but variably affected *IbCRY*.

## 3. Discussion

### 3.1. Effects of Deep Shading on Agronomic Traits and Leaf Morphology

The observed agronomic changes under 70% shading align with classic shade avoidance responses [11,24,25]. Plants exhibited increased plant height, vine length, and internode elongation—likely mediated through phytochrome-auxin signaling to enhance light foraging [24,26]. However, this elongation may compromise mechanical strength and raise lodging risk, reflecting a shift in resource allocation from secondary growth (thickening) to primary growth (elongation) under low light.

Leaf morphology also exhibited high plasticity. Increased leaf area represents a typical adaptation to enhance light capture, albeit often at the expense of reduced leaf thickness and photosynthetic efficiency per unit area [16,24]. Concurrent reduction in leaf dry matter indicates decreased net photosynthesis. Notably, shading induced a shift from lobed to more cordate leaf shapes—a morphological response seldom reported in other vegetables, which may further aid in light interception under deep shade.

### 3.2. Integrated Regulation of Leaf Coloration, Light Signaling, and Gene Expression

Chlorophylls absorb red and blue light but reflect green light, whereas anthocyanins absorb UV and blue-green light and reflect red light [11]. Shading significantly altered leaf pigment composition, increasing chlorophyll and carotenoid contents while suppressing anthocyanin accumulation. This resulted in darker green leaves with reduced red coloration—an adaptive strategy to maximize absorption of red and blue light through photosynthetic pigments while reducing reflectance via decreased anthocyanins [11,27].

At the molecular level, these changes were coordinated through light signaling pathways mediated by photoreceptors, including phytochromes (red light receptor), cryptochromes and photoprotection (blue light receptor), and UVR8 (UV-B receptor) [11,27,28]. Shading upregulated chlorophyll biosynthesis genes but downregulated anthocyanin-related genes. Notably, photoreceptor gene expression exhibited pigment-mediated feedback: *IbPHY* was elevated in red-hued varieties, potentially enhancing red light sensitivity under shade, whereas *IbUVR8* was higher in green/yellow types, possibly attenuating UV-B-induced anthocyanin synthesis. Concurrent suppression of *IbHY5*, a key positive regulator of photomorphogenesis, further supports shade-induced modulation of light signaling cascades. These results suggest a pigment–photoreceptor feedback loop that fine-tunes light capture and photoprotection under shaded conditions.

### 3.3. Shading-Induced Changes in Nutritional and Antioxidant Properties

Sweetpotato leaves are valued for their nutritional and antioxidant properties [6]. This study indicates that both leaf maturation and 70% shading reduce soluble sugar, phenolic compounds, flavonoids, and total antioxidant capacity. Additionally, shading also reduces soluble protein and cellulose in most varieties.

Young sweetpotato leaves generally exhibited higher antioxidant capacity than mature leaves. This pattern aligns with observations in trees: young leaves often possess underdeveloped physiological and photosynthetic structures and are exposed to intense light at the canopy top, thus requiring enhanced photoprotection [29].

Deep shading reduced key nutritional components—soluble sugars, soluble proteins, cellulose, phenolics, and flavonoids—leading to diminished total antioxidant capacity. These declines correlate with limited photosynthetic activity under low light, which restricts substrate and energy supply for metabolite synthesis [24,25,30]. As part of shade adaptation, plants prioritize resource allocation toward structural elongation and light-seeking growth over accumulation of defensive compounds.

The reduction in total antioxidant capacity was attributable to decreased levels of phenolics, flavonoids, and anthocyanins. Among the 16 agronomic and physiological parameters analyzed, antioxidant capacity correlated most strongly with soluble sugar and dry matter content, followed by TPC and TFC. Phenolics and flavonoids are key contributors to antioxidant activity [5,7,31], and their close link with agronomic traits underscores the interconnection between growth and metabolic adaptation. Soluble sugars in sweetpotato leaves—mainly glucose, fructose, and sucrose [15,16]—are key determinants of taste and quality. The strong correlation between soluble sugar content and antioxidant capacity may be attributed to their roles in stabilizing antioxidant enzymes via osmotic regulation [2] or involvement in environmental adaptation [32].

## 4. Materials and Methods

### 4.1. Plant Materials

Sweetpotato varieties were obtained from the National Tropical Plant Germplasm Resource Center and cultivated at the Batou Experimental Base (18.38° N, 109.15° E) in Sanya, Hainan, China, from 2023 to 2025. Twelve varieties with distinct leaf characteristics were used. Healthy vine cuttings were planted in organically fertilized soil on 15 January 2025. After one month of growth, all shoot tips were pruned to promote uniform new shoot development. Plants were then divided into two groups: natural light (CK) and 70% shading by shade cloth (deep shading), with three replicates each. After 20 days of treatment, agronomic traits were measured, and samples were collected for physiological and qRT-PCR analyses. Weather conditions during the treatment period, including temperature and light intensity (ranging from 29,950 to 111,983 Lux at midday), are provided in Table S1.

For sample collection, the apical vine tip was designated as developmental stage S1, with subsequent leaves labeled S2–S6 (Figure S2). Young leaves (Y) were pooled from S1 to S3 of 3–6 vines per replicate, and mature leaves (M) from S4 to S6. Four sample types were defined for each cultivar: YL (young leaves, natural light), YS (young leaves, 70% shading), ML (mature leaves, natural light), and MS (mature leaves, 70% shading). A total of 12 biological replicates were collected. Six replicates were immediately frozen in liquid nitrogen and stored at −80 °C for qRT-PCR and backup. Three fresh replicates were used for physiological assays on a fresh weight (FW) basis. The remaining three replicates were dried and ground into a fine powder for dry weight (DW)-based analyses. The drying procedure included enzyme deactivation at 105 °C for 20 min, followed by drying at 70 °C until constant weight. The dried material was then ground, passed through a 40-mesh sieve, and stored in airtight bags with desiccant at room temperature in a cool, dark place until further analysis.

### 4.2. Agronomic Traits

Leaf area was measured as the average area of S4–S6 leaves. Dry matter content was calculated as the ratio of dry weight to fresh weight. Plant height was defined as the vertical distance from the main stem base to the apical growing point. Maximum vine length was measured as the full length of the main stem. Stem thickness was assessed as the diameter at the base of the S6 leaf, and internode length as the average distance between adjacent nodes at S4–S6.

### 4.3. Colorimeter Measurements

Leaf color was measured on both sides of S1–S6 leaves using a portable colorimeter (CR-10 Plus, Konica Minolta, Osaka, Japan) calibrated with a white standard. CIEL*a*b* values were recorded under a pulsed xenon lamp (D65), with an 8 mm aperture, 8°/d illumination, a 10° observation angle, and 1 s measurement time. L* indicates lightness (0–100), a* represents green (−) to red (+), and b* indicates blue (−) to yellow (+). Three replicates were measured.

### 4.4. Measurement of Chlorophylls, Carotenoids, and Anthocyanins

Pigments were measured on FW basis.

Chlorophylls and carotenoids were extracted with 95% ethanol and quantified spectrophotometrically [17]. Briefly, the absorbance of the derived supernatants was measured at 665, 649, and 470 nm. Ca (mg/L) = 13.95A_665_ − 6.88A_649_, Cb (mg/L) = 24.96A_649_ − 7.32A_665_, and Cx (mg/L) = (1000A_470_ − 2.05Ca − 114.8Cb)/245. Chlorophyll a content (mg/gFW) = Ca × DR; chlorophyll b content (mg/gFW) = Cb × DR; carotenoid content (mg/gFW) = Cx × DR mg/gFW. In the above formulas, DR is the dilution rate.

Anthocyanins were extracted with 1% HCl-methanol and quantified as described by Shi et al. [7]. Total anthocyanin content (TAC) was calculated with Equation (1):

TAC (mg·g^−1^ FW) = (A_530_ − 0.25 × A_657_) × V × DR × MW/(ε × L × m) (1)

V is the extraction volume (mL); DR is the dilution rate; MW is the molecular weight (449.2 g mol^−1^ for cyanidin-3-glucoside); ε is the molar extinction coefficient (29,600 L mol^−1^·cm^−1^ for cyanidin-3-glucoside); L is the path length (1 cm) of the cuvette; and “m” is the fresh weight.

### 4.5. Determination of Soluble Protein, Soluble Sugar, and Cellulose Contents

Soluble protein and sugar content were determined on an FW basis, while cellulose was measured on a DW basis.

Soluble protein contents were quantified using Coomassie Brilliant Blue-based kits (A045-2, Nanjing Jiancheng, China). Absorbance was measured at 595 nm, and concentrations were calculated by comparison with a protein standard solution (0.524 g/L).

Soluble sugar contents were analyzed using plant soluble sugar assay kits (BC0035, Solarbio, Beijing, China) based on the anthrone colorimetric method. The constructed glucose standard curve (y = 5.2836x − 0.1281; R^2^ = 0.9857) was used for quantification.

Cellulose contents were determined with cellulose assay kits (BC4285, Solarbio). The procedure involved the removal of soluble sugars, hemicellulose, starch, and lignins, followed by acid hydrolysis to convert cellulose into glucose, which was then quantified indirectly via anthrone colorimetry [33]. Briefly, dried samples were treated with 80% ethanol, mixed, and incubated at 90 °C for 20 min. After cooling, the samples were washed sequentially with 80% ethanol and acetone, then dried. Subsequent rinses were performed three times with ddH_2_O and once with acetone, followed by drying. The samples were homogenized in ddH_2_O, and concentrated sulfuric acid was added slowly in an ice-water bath. After 30 min on ice, the supernatant was mixed with sulfuric acid and anthrone reagent, incubated at 95 °C for 10 min, and cooled. Absorbance was measured at 620 nm, and cellulose content was calculated using a glucose standard curve (y = 9.8477x + 0.0073; R^2^ = 0.9997).

### 4.6. Determination of TPC, TFC, and Antioxidant Capacity

The total phenolic contents (TPC) and total flavonoid contents (TFC) were measured on a DW basis, and the antioxidant capacities were assessed on an FW basis.

TPC was determined using the Folin–Ciocalteu method, with gallic acid (GAE) used as the quantification standard (y = 2.5966x + 0.0307; R^2^ = 0.9976). The TPC of the samples was expressed as milligrams of GAE equivalents per gram of leaf powder (mg GAE/g DW).

TFC was determined using a plant flavonoid assay kit (BC1335, Solarbio), following the aluminum chloride colorimetric method. A rutin standard curve (y = 0.6586x − 0.0484; R^2^ = 0.9732) was applied for calculation. The results were expressed as mg rutin equivalents per gram of leaf powder (mg rutin/g DW).

The total antioxidant capacity was assessed with a FRAP assay kit (BC1315, Solarbio), which measures the ferric reducing power. A standard curve (y = 12.045x − 0.0019; R^2^ = 0.9998) using FeSO_4_ was applied for quantification.

### 4.7. RNA Extraction and qRT-PCR

Total RNA was extracted using the RNAprep Pure Polysaccharide and Polyphenol Plant Kit (DP441, TIANGEN, Beijing, China). cDNA was synthesized from 2 μg RNA and diluted 4-fold for qRT-PCR. Reactions used a SYBR Green-based kit (AQ601-02, TransGen, Beijing, China) with *IbARF* as the reference gene. Melting curves were analyzed to confirm amplification specificity. Relative gene expression was calculated using the ΔCt method relative to the reference gene [34]. Primers are designed with the reannotated genome of *Ipomoea batatas* cv. ‘Taizhong 6’ [35], and listed in Table S2.

### 4.8. Statistical Analysis

All measurements were performed in triplicate. Data are presented as mean ± standard error (SE). Statistical analysis was performed using IBM SPSS Statistics 27 (SPSS Inc., Chicago, IL, USA). For agronomic traits, differences between CK and 70% shading treatments were assessed by *t*-test, with * *p* < 0.05 and ** *p* < 0.01. For physiological and qRT-PCR data, differences among the four sample types within each variety were analyzed by one-way ANOVA at *p* < 0.05. Pearson’s correlation analysis was conducted among the 16 agronomic and physiological parameters, all of which satisfied the normality assumption. Differences in significance were tested by *t*-test, with *p* < 0.05 (*), *p* < 0.01 (**), and *p* < 0.001 (***).

## 5. Conclusions

Sweetpotato leaves exhibit considerable plasticity in agronomic, nutritional, and antioxidant properties in response to developmental stage and light environment. Leaf maturation consistently increased chlorophyll content but decreased soluble sugars, phenolics, flavonoids, and antioxidant activity. No clearly shade-tolerant cultivar was identified among the 12 tested varieties. Under 70% shading, plants displayed enhanced leaf expansion and elongation traits, but reduced dry matter, sugar, cellulose, and antioxidant capacities. Leaf morphology shifted from lobed to more cordate, accompanied by darker green and less red coloration resulting from elevated chlorophyll and suppressed anthocyanin accumulation. Antioxidant capacity was most strongly correlated with soluble sugar and dry-matter content, followed by TPC and TFC, underscoring the linkage between growth and metabolic traits. At the molecular level, shading upregulated chlorophyll-related genes but downregulated anthocyanin biosynthetic genes. Light-signaling responses appeared modulated by pigment feedback, with photoreceptors showing leaf color-specific expressions: *IbPHY* was higher in red-hued varieties, while *IbUVR8* was elevated in yellow/green varieties. *IbHY5* was suppressed by shading. These findings illustrate integrated phenotypic and physiological adaptations to deep shading, supporting breeding and cultivation of vegetable sweetpotato. Future studies should employ finer light gradients on elite cultivars to determine optimal light intensity for production.

## Figures and Tables

**Table 1:** Effects of deep shading on aboveground agronomic indices in the 12 sweetpotato varieties.

Variety	Usage	Treatment	Leaf Area (cm^2^)		Dry Matter (%)		Plant Height (cm)		Vine Length (cm)		Stem Thickness (mm)		Branch Number		Internode Length (cm)	
			Value	Change	Value	Change	Value	Change	Value	Change	Value	Change	Value	Change	Value	Change
Fu18	V	L	35.33 ± 0.66	156% **	11.50 ± 0.12	83.6% **	22.03 ± 0.79	116% **	27.38 ± 1.42	143% **	6.97 ± 0.44	82%	5.33 ± 0.33	50% **	1.59 ± 0.11	130% **
		S	55.07 ± 2.70		9.61 ± 0.39		25.48 ± 0.58		39.13 ± 3.46		5.73 ± 0.41		2.67 ± 0.33		2.07 ± 0.10	
HD-V4	V	L	31.39 ± 3.62	119%	11.07 ± 0.10	81.1% **	24.61 ± 0.54	113% **	26.04 ± 1.14	142% **	6.80 ± 0.26	69% **	4.33 ± 0.67	77%	2.04 ± 0.13	142% **
		S	37.32 ± 2.06		8.98 ± 0.23		27.74 ± 0.53		36.93 ± 2.22		4.70 ± 0.28		3.33 ± 0.33		2.89 ± 0.20	
HD-V7	V&R	L	34.09 ± 1.60	123% **	11.60 ± 0.06	82.2% **	24.16 ± 0.62	102%	39.41 ± 2.34	129% **	4.03 ± 0.20	91%	3.33 ± 0.33	100%	2.78 ± 0.22	174% **
		S	41.84 ± 0.44		9.53 ± 0.19		24.53 ± 0.42		50.99 ± 1.52		3.68 ± 0.26		3.33 ± 0.33		4.83 ± 0.16	
RXC1	V	L	53.27 ± 1.46	120% *	10.77 ± 0.24	86.3% *	21.19 ± 0.20	131% **	33.48 ± 2.10	130% **	5.58 ± 0.25	79% *	3.67 ± 0.67	73%	2.33 ± 0.10	119%
		S	64.11 ± 2.06		9.29 ± 0.26		27.70 ± 0.80		43.62 ± 1.95		4.43 ± 0.34		2.67 ± 0.33		2.77 ± 0.30	
Golden	O	L	47.74 ± 0.74	159% **	11.78 ± 0.19	80.0% **	13.72 ± 0.22	125% **	25.89 ± 1.14	133% *	4.83 ± 0.17	99%	3.67 ± 0.67	91%	1.61 ± 0.06	128% **
		S	75.66 ± 2.77		9.42 ± 0.10		17.18 ± 0.86		34.40 ± 2.54		4.78 ± 0.20		3.33 ± 0.67		2.06 ± 0.11	
Ayamurasaki	R	L	44.20 ± 1.61	149% **	10.08 ± 0.28	84.7% **	16.87 ± 0.70	134% **	37.69 ± 3.32	140% **	5.67 ± 0.27	86% *	4.33 ± 0.67	100%	1.81 ± 0.09	155% **
		S	65.93 ± 3.02		8.54 ± 0.01		22.62 ± 1.60		52.63 ± 2.39		4.85 ± 0.17		4.33 ± 0.33		2.81 ± 0.11	
Su24	R	L	40.73 ± 1.38	168% **	10.60 ± 0.15	100.2%	22.68 ± 0.78	116% *	28.25 ± 2.03	188% **	5.65 ± 0.11	87%	2.00 ± 0.00	184% *	2.28 ± 0.06	169% **
		S	68.55 ± 1.63		10.62 ± 0.18		26.35 ± 0.96		53.11 ± 1.02		4.93 ± 0.31		3.67 ± 0.67		3.86 ± 0.18	
Fu202	R	L	44.50 ± 2.86	134% *	14.15 ± 0.08	73.9% **	18.12 ± 0.75	113%	33.99 ± 1.50	130% *	5.07 ± 0.29	82% *	4.00 ± 0.58	75%	2.84 ± 0.07	134% **
		S	59.62 ± 1.84		10.46 ± 0.08		20.55 ± 0.80		44.31 ± 4.91		4.17 ± 0.25		3.00 ± 0.58		3.81 ± 0.09	
HD7791	V&O	L	68.57 ± 1.25	118% *	14.09 ± 0.34	74.1% **	22.01 ± 0.56	131% **	38.69 ± 1.81	162% **	5.95 ± 0.18	82% **	5.00 ± 0.58	73%	3.04 ± 0.15	135% **
		S	80.72 ± 2.96		10.44 ± 0.03		28.73 ± 0.94		62.57 ± 6.18		4.88 ± 0.22		3.67 ± 0.67		4.09 ± 0.23	
Chuzi1	R	L	50.55 ± 0.78	143.5% **	14.35 ± 0.13	83.7% **	21.32 ± 0.40	129.1% **	54.12 ± 3.30	111%	6.00 ± 0.22	91%	4.00 ± 0.00	83%	4.59 ± 0.28	122% *
		S	72.53 ± 1.32		12.01 ± 0.10		27.53 ± 1.04		60.12 ± 4.63		5.48 ± 0.30		3.33 ± 0.33		5.60 ± 0.27	
GCS12-557	V	L	29.14 ± 0.37	178% **	10.26 ± 0.24	82% **	20.15 ± 0.84	124% **	21.17 ± 1.05	152% **	5.78 ± 0.24	86%	4.33 ± 0.88	85%	1.33 ± 0.05	147% **
		S	52.00 ± 1.70		8.45 ± 0.10		25.06 ± 0.94		32.19 ± 2.47		4.97 ± 0.31		3.67 ± 0.67		1.96 ± 0.09	
Fu23	V	L	44.21 ± 1.75	168% **	12.16 ± 0.21	86% **	23.03 ± 0.49	103%	36.75 ± 4.00	124%	5.52 ± 0.15	87% **	3.33 ± 0.33	140%	2.33 ± 0.24	189% **
		S	74.30 ± 2.24		10.50 ± 0.25		23.82 ± 0.20		45.42 ± 1.10		4.82 ± 0.15		4.67 ± 0.67		4.41 ± 0.17	

Note: Treatment: L = Under natural light (CK), S = Under 70% shading; Usage: V = Vegetable, R = Tuberous Root, and O = Ornamental. Change is the percentage of S/L, and the significance of differences between CK and 70% shading was tested by *t*-test, with *p* < 0.05 (*) and *p* < 0.01 (**).

## Data Availability

All data generated or analyzed during this study are included in this published article and its Supplementary Information files.

## Supplementary Materials

The following supporting information can be downloaded at: https://www.mdpi.com/article/10.3390/plants14192969/s1, Figure S1: The 12 tested sweetpotato varieties, categorized into four leaf color series; Figure S2: Criteria for leaf developmental stages S1–S6 in sweetpotato; Figure S3: Leaf morphology at stages S1–S6 in the 12 sweetpotato varieties; Figure S4: CIEL*a*b* parameters for leaves from S1 to S6 of the 12 sweetpotato varieties; Figure S5: Dissolution curves of amplified products exhibited good specificity in qRT-PCR amplifications; Table S1: Weather conditions and illuminance data during the 20-day treatment period; Table S2: Primers used in qRT-PCR.

## Abbreviations

The following abbreviations are used in this manuscript:

YL	Young leaves under natural light
YS	Young leaves under 70% shading
ML	Mature leaves under natural light
MS	Mature Leaves under 70% Shading
FW	Fresh weight
DW	Dry weight
TPC	Total phenolic contents
TFC	Total flavonoid contents
SPL9	Squamosa promoter binding protein-like 9
HEMB	Heme biosynthesis gene B
PORC	Protochlorophyllide oxidoreductase C
CAO	Chlorophyll a oxygenase
PAL	Phenylalanine ammonia-lyase
CHS	Chalcone synthase
PHY	Phytochrome
CRY	Cryptochrome
UVR8	UV resistance locus 8
HY5	Elongated hypocotyl 5
TF	Transcription factor
