# Supplementary material for: Effects of Deep Shading on Agronomic Traits, Coloration, and Antioxidant Properties in Sweetpotato Leaves
Source: Plants (Basel). 2025 Sep 25;14(19):2969. doi: 10.3390/plants14192969 (PMC12526451; doi:10.3390/plants14192969)
Supplement: Supplementary file 1 [file plants-14-02969-s001.zip › plants-3862374-supplementary.pdf]

Figure S1

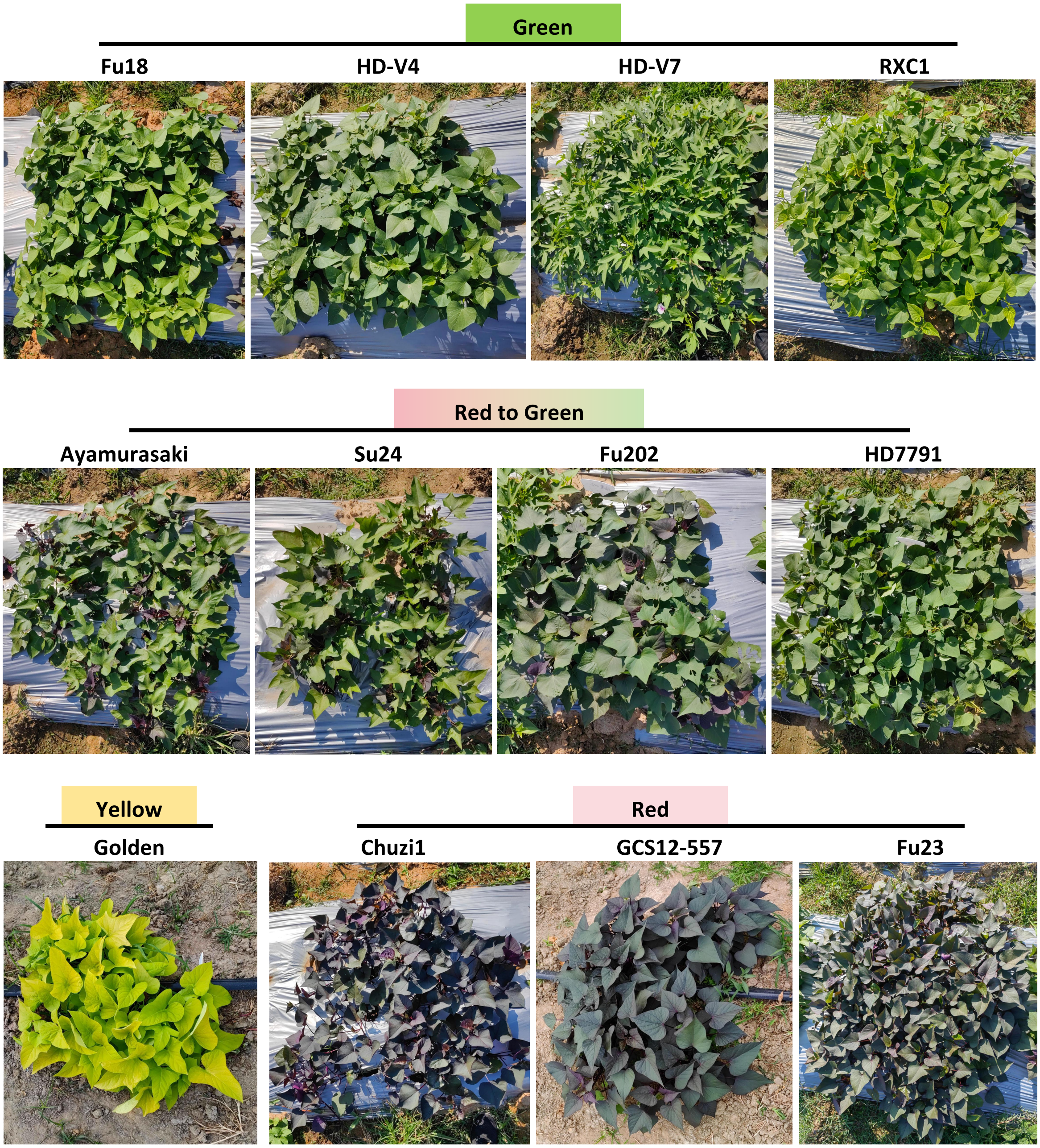

Figure S1. The 12 tested sweetpotato varieties, categorized to four leaf color series.

Figure S2

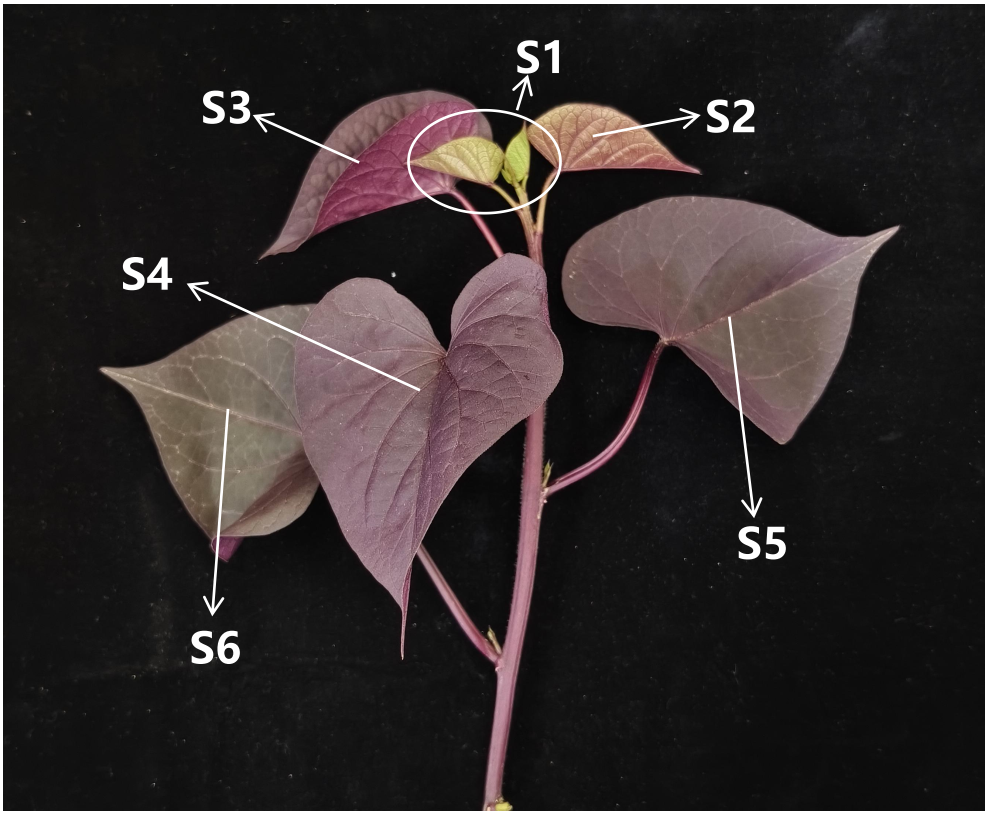

Figure S2. Criteria for leaf developmental stages S1-S6 in sweetpotato. Young leaves were pooled from S1-S3, and mature leaves were pooled from S4-S6. Exemplified by the vine of ‘Chuzi1’.

Figure S3

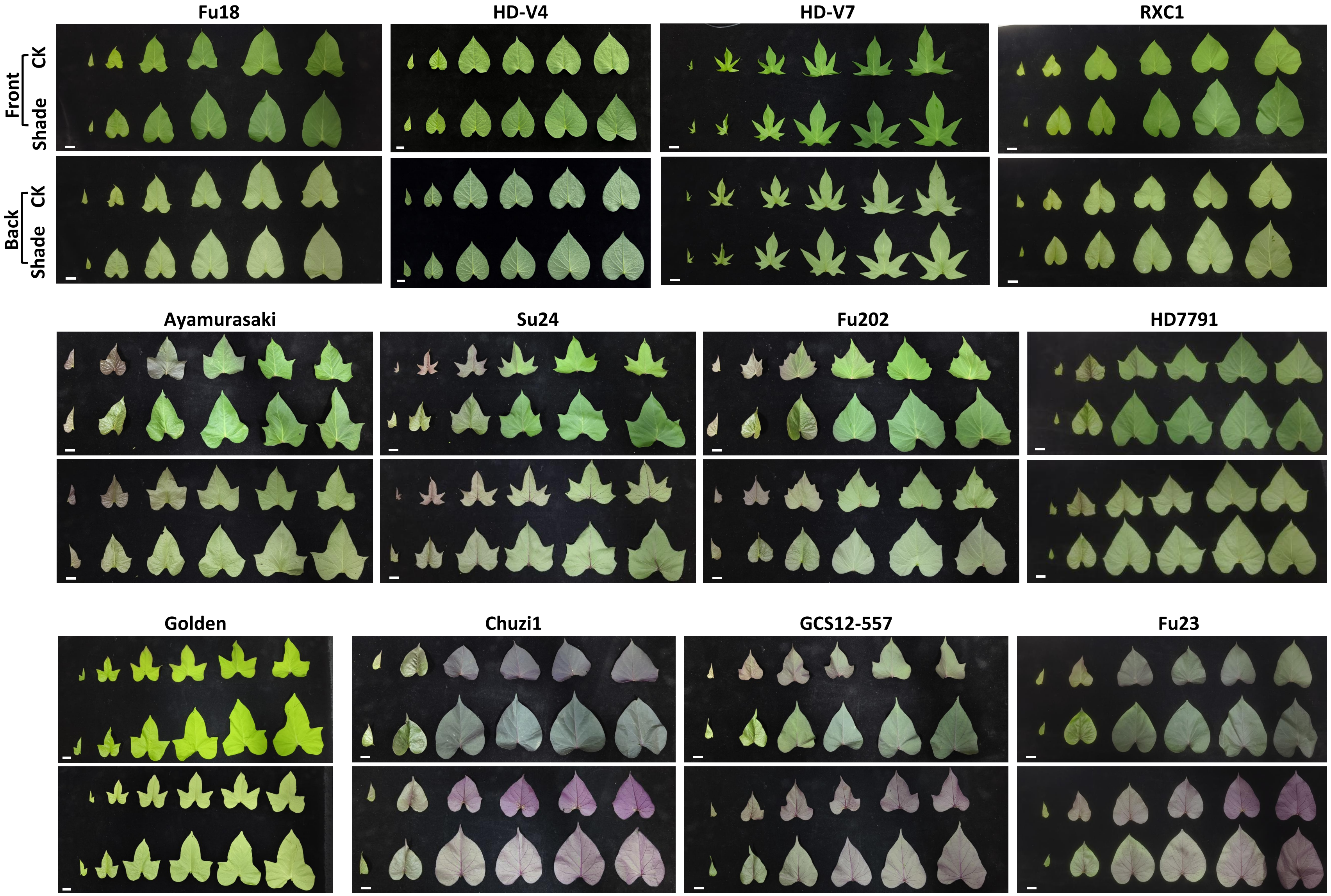

Figure S3. Leaf morphology at stages S1-S6 in the 12 sweetpotato varieties. Bar=1 cm. The front represents the adaxial surface of the leaves, and the back represents the abaxial surface.

Figure S4

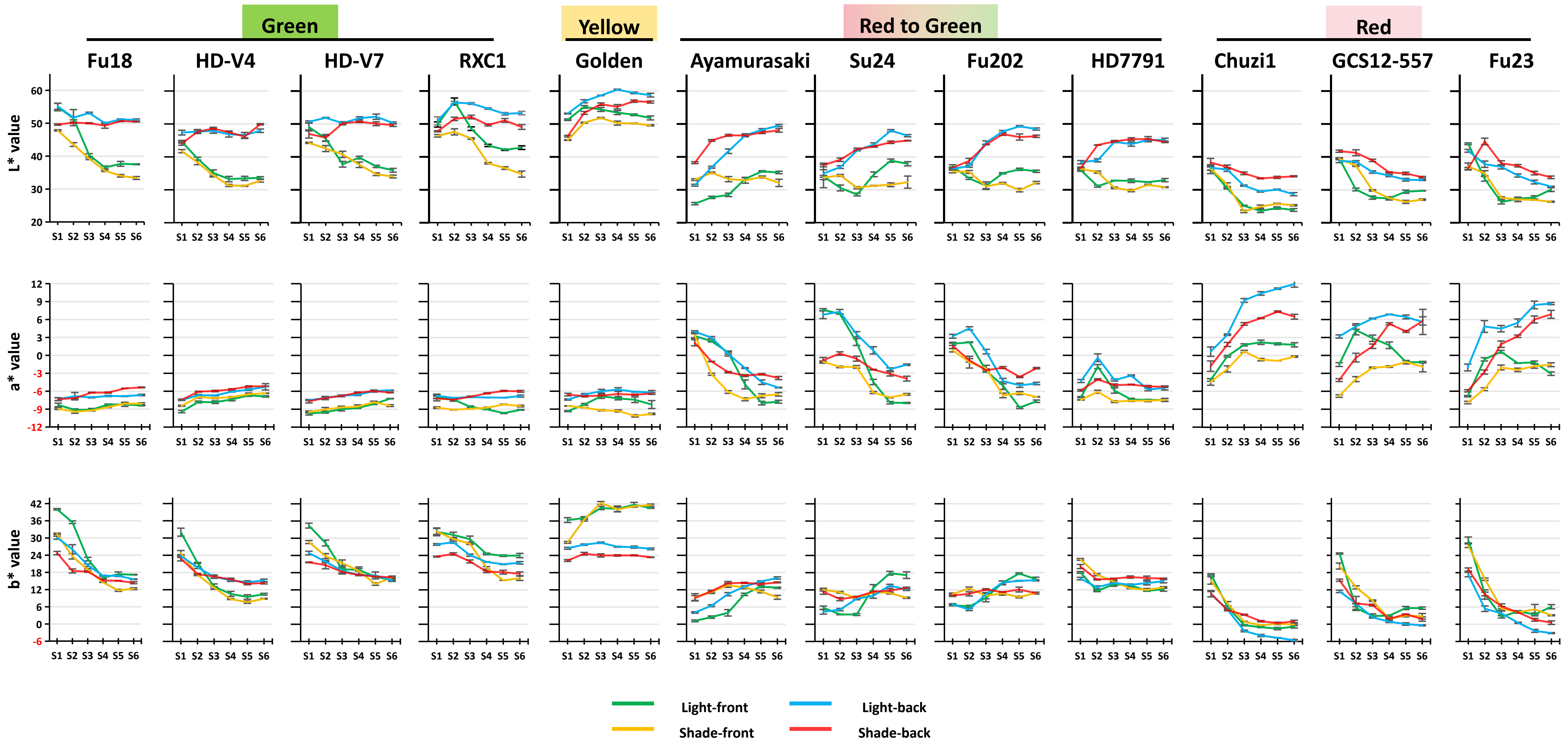

Figure S4. CIEL*a*b* parameters for leaves from S1 to S6 of the 12 sweetpotato varieties.

Figure S5

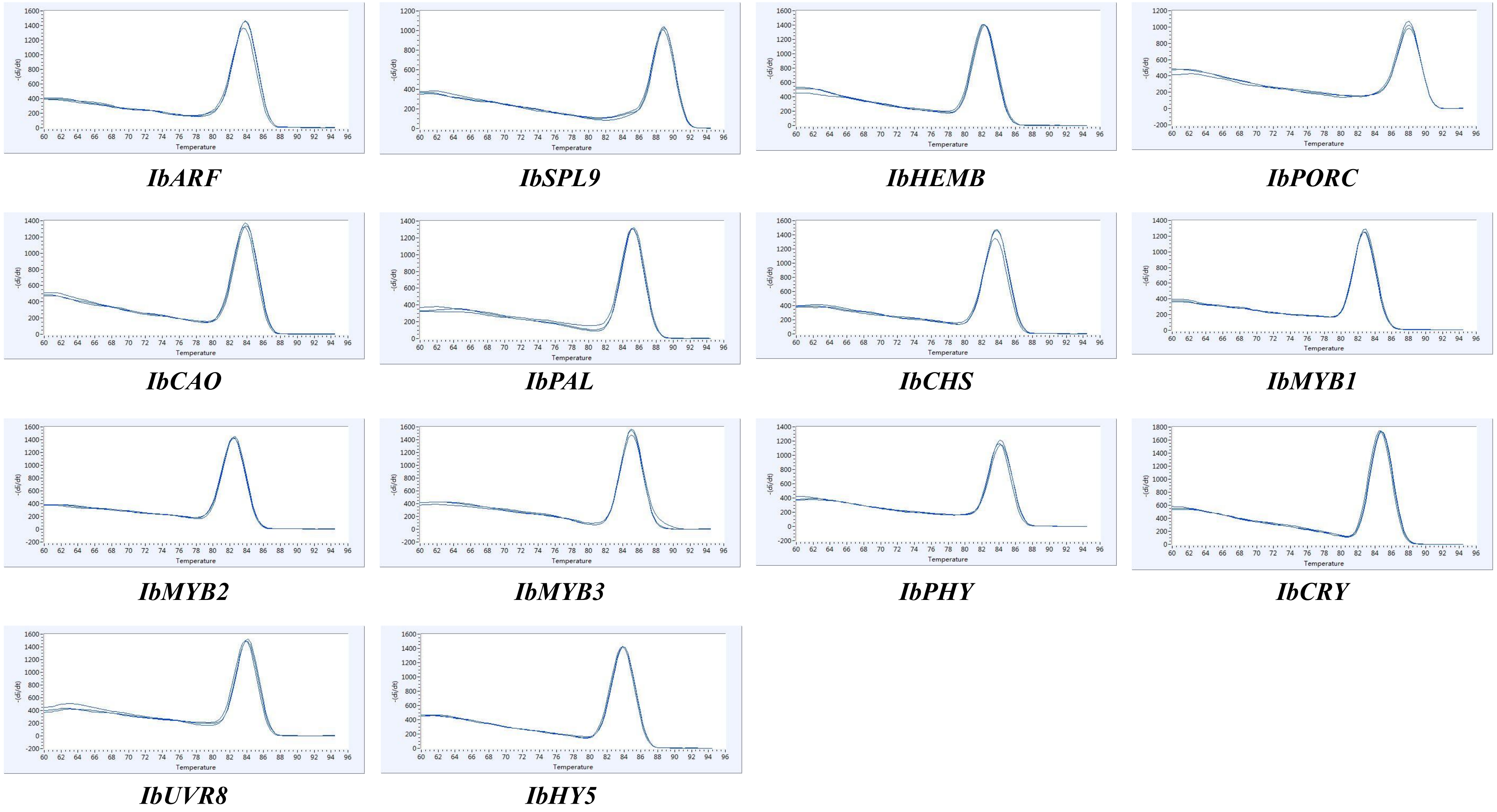

Figure S5. Dissolution curves of amplified products exhibited good specificity in qRT-PCR amplifications.

Table S1. Weather conditions and illuminance data during the 20-day treatment period.

Day	Date	T_{Min}	T_{Max}	Weather	Illuminance (Lux)
1	2025/2/11	16℃	24℃	Cloudy	31648
2	2025/2/12	20℃	27℃	Light rain/Cloudy	87730
3	2025/2/13	17℃	25℃	Sunny/Cloudy	50532
4	2025/2/14	18℃	27℃	Cloudy	48427
5	2025/2/15	19℃	29℃	Sunny/Cloudy	94192
6	2025/2/16	18℃	29℃	Sunny	111983
7	2025/2/17	19℃	28℃	Sunny/Cloudy	99550
8	2025/2/18	17℃	26℃	Sunny/Cloudy	45993
9	2025/2/19	16℃	23℃	Sunny/Cloudy	88010
10	2025/2/20	17℃	25℃	Sunny	101983
11	2025/2/21	18℃	25℃	Sunny/Cloudy	81168
12	2025/2/22	19℃	25℃	Cloudy	50555
13	2025/2/23	17℃	25℃	Sunny/Cloudy	95775
14	2025/2/24	17℃	22℃	Sunny/Cloudy	96570
15	2025/2/25	17℃	24℃	Cloudy	42870
16	2025/2/26	18℃	25℃	Cloudy	45460
17	2025/2/27	16℃	27℃	Sunny/Cloudy	92337
18	2025/2/28	17℃	28℃	Cloudy	55322
19	2025/3/1	18℃	28℃	Cloudy	29950
20	2025/3/2	17℃	31℃	Sunny/Cloudy	89008

Note: Fields located at the Batou Experimental Base (N 18.38°, E 109.15°), Sanya, Hainan, China. The measurement time was daily at 12:30 noon. T_{Min} and T_{Max} represent the minimum and maximum temperatures for the day, respectively.

Table S2. Primers used in qRT-PCR.

Genes	Primer names	primer sequences (5'-3') *
IbARF	ARF-F	CTTTGCCAAGAAGGAGATGC
	ARF-R	TCTTGTCCTGACCACCAACA
IbMYB1	MYB1-F	GGCAACAGGTGGTCGCTTAT
	MYB1-R	CCGTGATTTCTGGGGCTTT
IbMYB2	MYB2-F	AGAAGTATGGGGAAGGGAAATG
	MYB2-R	TGCCTAAGAGCCGATGGAG
IbMYB3	MYB3-F	GCTTATCGCCGGTAGAAT
	MYB3-R	CACTGGTAACAATGGTCGT
IbPAL（IB06G26590）	PAL-F	TTCATCCCGCAAGGTTTTC
	PAL-R	CGGTGTTCTTCTCATTCTCCC
IbCHS	CHS-F	GTCAACCAAAGCACCTACCC
	CHS-R	CTCCACCACCGCAATGTC
IbPHY（IB04G06080）	PHY-F	TGAAGACGCTGAAAGTGTATGG
	PHY-R	CGGGGCAGATAAACCTGAA
IbCRY（IB02G25730）	CRY-F	AAGAAGAGGAAAGGCCGGTAA
	CRY-R	CGGGAACAGAAAACGAATGAC
IbUVR8（IB05G28880）	UVR8-F	TATTCTTGGGGTAGAGGCACA
	UVR8-R	TCATCTGGAACAACTGCGTATC
IbHY5（IB11G21060）	HY5-F	CGATGAGATCAGAAGAGTGCC
	HY5-R	CTTGCTGCGCCGAAACT
IbSPL9（IB06G23690）	SPL9-F	GGAGAATGTTTCGCTGGAGTT
	SPL9-R	TGGGAGGCAGAATGGTGA
IbPORC（IB01G02810）	PORC-F	AAGGCGTACAAGGACAGCAA
	PORC-R	AGGCGGAACAAGGGGAT
IbCAO（IB04G11990）	CAO-F	GGGGCAGAGTACGAAGCAGT
	CAO-R	CCCAATTACAAGCCGCAGA
IbHEMB（IB02G19560）	HEMB-F	CCTCGAACTATACGACTGCTAAAA
	HEMB-R	TGCTCTGGCTTGGCTGACT

Note: Primers for *IbARF*, *IbMYB1*, *IbMYB2*, *IbMYB3*, *IbCHS* are from Deng et al. [17] and Wang et al. [23]. Primers for other genes are designed with the the reannotated genome of *Ipomoea batatas* cv. 'Taizhong 6' (https://www.sweetpotao.com/download_genome.html)[35].
